# An *SNX31* variant underlies dominant familial exudative vitreoretinopathy-like pathogenesis

**DOI:** 10.1172/jci.insight.167032

**Published:** 2023-05-22

**Authors:** Ningda Xu, Yi Cai, Jiarui Li, Tianchang Tao, Caifei Liu, Yan Shen, Xiaoxin Li, Leiliang Zhang, Mingwei Zhao, Xuan Shi, Jing Li, Lvzhen Huang

**Affiliations:** 1 Department of Ophthalmology, Peking University People’s Hospital, Eye Diseases and Optometry Institute, Beijing Key Laboratory of Diagnosis and Therapy of Retinal and Choroid Diseases, College of Optometry, Peking University Health Science Center, Beijing, China.; 2 Beijing Key Laboratory of DNA Damage Response, College of Life Sciences, Capital Normal University, Beijing, China.; 3 Key Laboratory of Cell Proliferation and Differentiation of Ministry of Education, College of Life Sciences, Peking University, Beijing, China.; 4 Department of Ophthalmology, Xiamen Eye Center of Xiamen University, Xiamen, China.; 5 Institute of Basic Medicine, Shandong First Medical University & Shandong Academy of Medical Sciences, Jinan, China.

**Keywords:** Genetics, Ophthalmology, Genetic diseases, Genetic variation

## Abstract

Familial exudative vitreoretinopathy (FEVR) is a complex hereditary eye disorder characterized by incomplete development of the retinal vasculature, which thereby affects retinal angiogenesis. But the genetic factors contributing to FEVR’s development or pathogenesis remain elusive. In a Chinese family with FEVR with 19 members, by using whole-exome sequencing, we identified a candidate disease-causing DNA variant in sorting nexin 31 (*SNX31*) (c.963delG; p. Trp321Cys), which results in a frameshift mutation. We studied the biochemical mechanism of this mutation and determined that it is deficient in β1-integrin binding and stability. The *SNX31* c.963delG point mutation mouse model (*SNX31^m/m^*) was constructed with CRISPR/Cas9 technology. At 2–4 months of age, *SNX31^m/m^* mice showed fundus phenotypes similar to FEVR-like changes, including vascular leakage and retinal atrophy. Moreover, we found that VEGF and apoptotic pathways were involved in these ocular phenotypes. Hence, our study extended the FEVR mutation spectrum to include *SNX31*. These findings expanded our understanding of the molecular pathogenesis of FEVR and may facilitate the development of methods for the diagnosis and prevention of FEVR.

## Introduction

Familial exudative vitreoretinopathy (FEVR) was first proposed and named by Criswick and Schepens in 1969 (1). It is hereditary and leads to retinal detachment and blindness in adults. It has been reported that, in children with retinal detachment, the incidence of FEVR is as high as 16% (2, 3), and 21%–64% of patients with FEVR have retinal detachment (4).

The clinical manifestations of FEVR are heterogeneous, occurring mostly at or after birth and generally involve both eyes simultaneously. The fundus is characterized by incomplete vascularization of the peripheral retina and a series of complications, including retinal traction and exudation, vitreous hemorrhage, and retinal detachment (4, 5). Currently, laser and surgery are the main treatments for FEVR. However, because of the distinctive anatomy of the child’s eye and the severe side effects of surgery, the surgical complications are quite serious. Therefore, early screening, early diagnosis, and timely treatment could greatly reduce the blindness rate of FEVR.

FEVR pathogenic genes identified thus far include zinc finger protein 408 (*ZNF408*) (6), kinesin family member 11 (*KIF11*) (7), catenin β 1 (*CTNNB1*) (8), frizzled 4 (*FZD4*) (9), LDL receptor–related protein 5 (*LRP5*) (10), tetraspanin 12 (*TSPAN12*) (11), and Norrie disease product (*NDP*) (12, 13). All these genes, except for *KIF11* and *ZNF408*, are directly or indirectly involved in FEVR pathogenesis through the Wnt/Norrin β-catenin signaling pathway. FEVR is a complex disease, and these pathogenic genes account for less than 50% of FEVR cases (6), suggesting that there may be unknown genes.

We encountered a Chinese family with FEVR without any of the known FEVR genes. Three members of this family were selected for whole-exome sequencing, and the potential mutation sites were validated by Sanger sequencing. We identified a frameshift heterozygous mutation in the sorting nexin 31 (*SNX31*) gene on chromosome 8, which truncates its C-terminal 4.1/ezrin/radixin/moesin (FERM) domain. Initially discovered by Carlton et al (14), sorting nexins (SNXs) are a group of proteins containing the phagocytic oxidase homology domain (Phox-homology domain [PX domain]) and FERM-like domain. The PX domain is a phospholipid-binding domain. By binding phospholipids and their corresponding phosphorylated derivatives, the target protein can be localized to the phospholipid-rich membrane, enabling it to perform its function (15). SNX31 binds the cytoplasmic domains of β1-integrin through its FERM domain (16). Integrin is essential in angiogenesis and vascular integrity and is the main mediator of vascular cell adhesion and migration through the extracellular matrix (17). In addition, a study found that angiogenesis dysfunction can cause retinal dystrophies (18), and integrin-knockout mice suffered from impaired retinal vascularization, local peripheral retinal hemorrhage, and deep retinal vascular sprouting defects (19). These findings have many similarities with the fundus phenotype of patients with clinical FEVR, who manifest abnormal development of retinal blood vessels (4).

We carried out a functional analysis of the *SNX31* mutation in the family with FEVR and found that its binding with β1-integrin is defective, suggesting that SNX31 may contribute to FEVR pathogenesis via β1-integrin. Furthermore, the *SNX31* variant affects human retinal microvascular endothelial cell (HRMEC) tube formation, cell migration, and proliferation. Moreover, we constructed an *SNX31* c.963delG point mutation mouse model, and it exhibited ocular phenotypes and inheritance patterns similar to those of FEVR. Based on molecular experiments and animal models, our study not only reveals a potentially new genetic mutation that leads to FEVR but also suggests that integrin, VEGF, and apoptosis pathways take part in ocular vascular disease development.

## Results

### Whole-exome sequencing revealed an SNX31 mutation in a FEVR pedigree.

**Figure 1:**
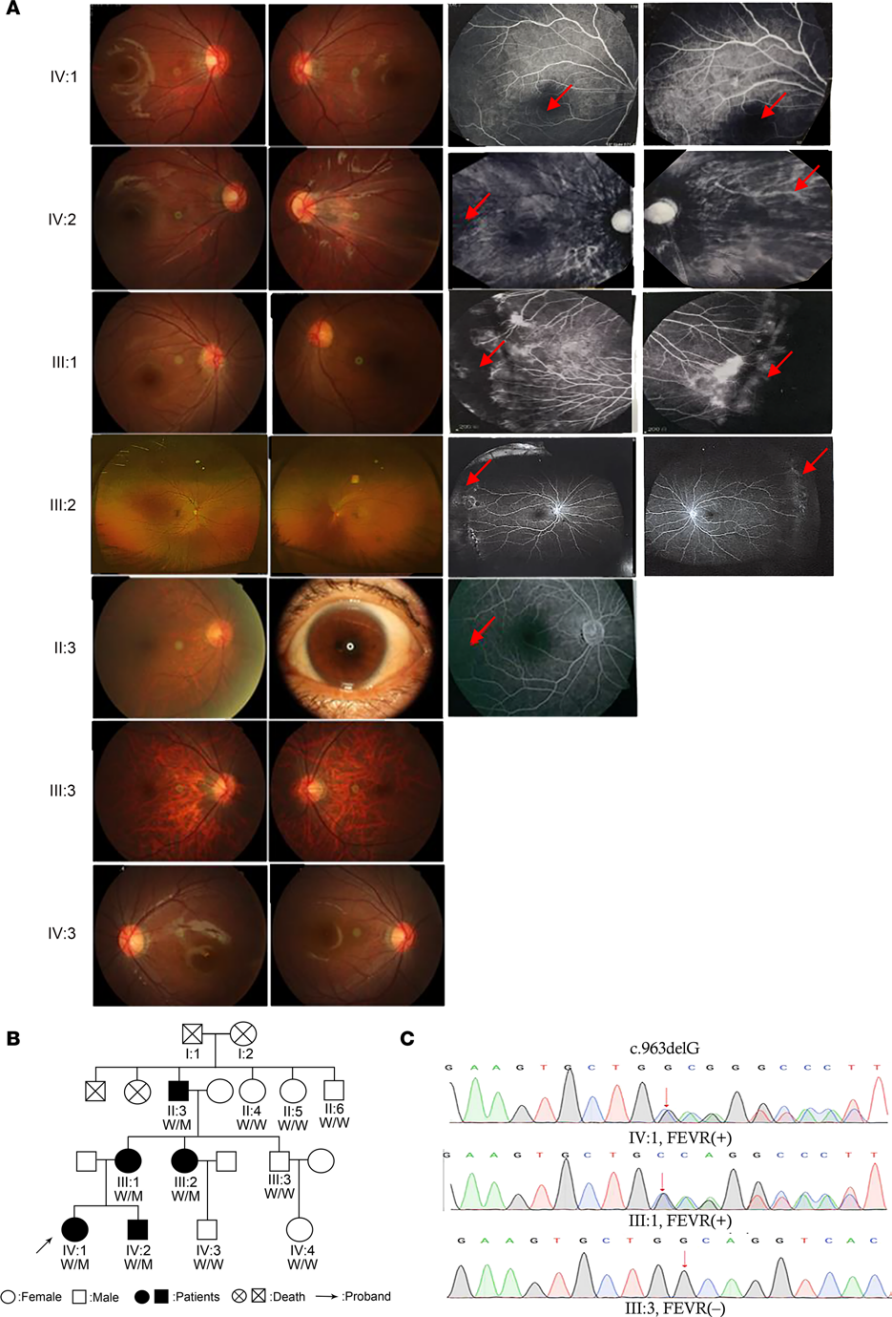
A mutation in *SNX31* was identified in a Chinese family with FEVR. (**A**) Fundus photography and fluorescence angiographies of patients in this family. IV:1, IV:2, III:1, III:2, and II:3 had FEVR; III:3 and IV:3 did not. The left eye of II:3 had pupillary seclusion, and so we could not perform fundus photography. The red arrows indicate no perfusion area of blood vessels. (**B**) FEVR pedigree. Whole-exome sequencing analysis and putative pathogenic mutation screening. The arrow indicates the IV:1 patient of this family. (**C**) Mutation identification by Sanger sequencing. Shown are the sequencing results of IV:1 (heterozygous mutant), III:1 (heterozygous mutant), and III:3 (WT). M, mutant; W, wild.

In an effort to identify new genes contributing to ocular diseases, we encountered a FEVR pedigree. There are 19 people in a 4-generation family, and 5 affected individuals were identified: a 6-year-old girl, IV:1 (proband), and her brother, IV:2; mother, III:1; mother’s sister, III:2; and grandfather, II:3 (Table 1). The patient’s fundus photograph and fluorescein angiography (FFA) were examined to confirm the diagnosis (Figure 1A).

The proband of this family with FEVR was a 6-year-old girl (IV:1), a full-term infant with no history of oxygen inhalation and low body weight. At birth, no abnormalities were found. At the age of 3 months, the patient tilted her head when gazing; at the age of 4 months, her left eyeball was slightly smaller and esotropic. At about 5 years old, the patient was prone to falling and bumping when running. After that, the patient went to the hospital for visual acuity and fundus examination, and amblyopia and retinal avascular area were found. Genetic testing of this family revealed no known mutations of the FEVR disease–causing genes, and therefore whole-exome sequencing was carried out on IV:1, IV:2, and III:1. A heterozygous and dominant variant (c.963delG) with an SNP of exon 8 in the *SNX31* gene was identified in all 3 patients, resulting in a frameshift from amino acid 321, thus generating a shortened protein of 342 amino acids. The mutation resulted in a truncated version of the FERM domain and was named SNX31-Mut afterward.

Sanger sequencing confirmed the mutation and cosegregation analysis (Figure 1, B and C). The c.963delG variant of *SNX31* was confirmed to be dominant in this family with FEVR (pedigree IV:1, III:1), and the uncle of patient IV:1 has a wild-type (WT) gene and a normal phenotype (pedigree III:3). Therefore, the c.963delG variant in the *SNX31* gene cosegregated with the disease phenotype in the family members tested. The variant did not exist in 1,000 normal, ethnically matched controls (data not shown). We concluded that mutations in the family with FEVR pedigree are completely cosegregated.

### Mutant SNX31 failed to bind β1-integrin, downregulated β1-integrin levels, and affected the basic function of vascular cells.

**Figure 2:**
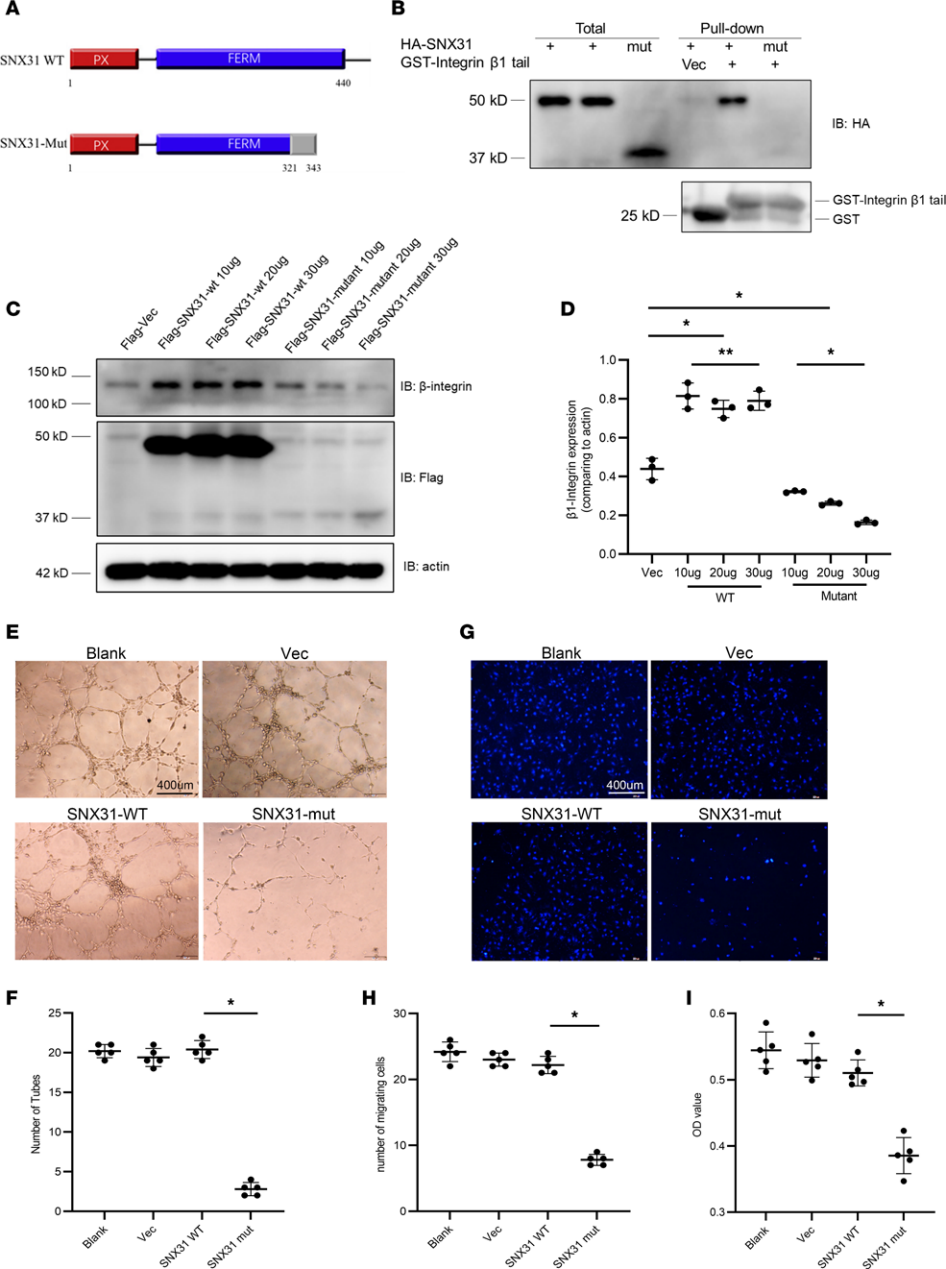
Mutant SNX31 failed to bind β1 integrin, downregulated β1 integrin levels, and affected the basic function of vascular cells. (**A**) A diagram showing the SNX31 structure, including the PX and FERM domain. The SNX31-mut we identified has a frameshift mutation at 321, resulting in a truncated protein of 343 amino acids. (**B**) 293T cells were transfected with the HA-SNX31-WT and HA-SNX31-Mut. GST-integrin β1 tail pulled down HA-SNX31-WT but not the HA-SNX31-Mut. (**C**) SNX31-WT increased the levels of β1-integrin while the SNX31 mutant decreased the levels of β1-integrin. Actin was used as a reference. (**D**) Quantitation of results in **C**. (**E** and **F**) The tube-forming ability of the SNX31-Mut in HRMECs was examined. Scale bar: 400 μm. (**G** and **H**) The migration ability of SNX31-Mut group is significantly less than the blank control, Flag-vec, and SNX31-WT groups. Scale bar: 400 μm. (**I**) Cell proliferation in the SNX31-Mut group was inhibited. The 3 transfection groups were compared with the blank group. Statistical analysis was performed via unpaired parametric *t* test or 1-way ANOVA. Data are shown as mean ± SEM. *P < 0.05, **P > 0.05. See complete unedited blots in the supplemental material.

Because the SNX31-Mut caused a frameshift in the FERM domain (Figure 2A), which is critical for β1-integrin interaction, we explored the potential underlying mechanism. The glutathione S-transferase–integrin (GST-integrin) tail was used to pull down HA-SNX31-WT and HA-SNX31-Mut, and the biochemical results showed that the integrin tail could bind with HA-SNX31-WT but not with HA-SNX31-Mut (Figure 2B). Because the binding between SNX31 and β1-integrin mediates the surface stability of β1-integrin, we examined β1-integrin protein levels. When we overexpressed Flag-SNX31-Mut in the cell, we found that it caused a decrease in β1-integrin levels in a dose-dependent manner (Figure 2, C and D), suggesting that Flag-SNX31-Mut is defective in integrin stability. However, according to the current experimental results, the specific mechanism cannot be confirmed, and both haploinsufficient and dominant negative mutations are possible.

To study the effect of mutant SNX31 protein on the angiogenesis ability of human HRMECs, we performed tube formation experiments. We transfected HRMECs with Flag-vec, Flag-SNX31-WT, and Flag-SNX31-Mut plasmids and then examined the cells under the microscope to determine the number of lumens. The results showed that the tube-forming ability of HRMECs transfected with Flag-vec and Flag-SNX31-WT was not statistically different from the blank control group (*P* > 0.05). However, HRMECs transfected with Flag-SNX31-Mut displayed little luminal morphology, and the tube-forming ability was significantly lower than that of the other groups (*P* < 0.05) (Figure 2, E and F).

To study the effect of SNX31-Mut on the migration ability of HRMECs, we conducted Transwell chamber assays. The migration ability of the SNX31-Mut group was lower than that of other groups (*P* < 0.05) (Figure 2, G and H).

We also used the Cell Counting Kit-8 (CCK-8 kit) to measure cell proliferation (Figure 2I). Compared with other groups, the proliferation of HRMECs in the SNX31-Mut transfection group was significantly inhibited (*P* < 0.05). Taken together, mutant SNX31 has an adverse effect on HRMEC tube formation, cell migration, and proliferation.

### Validation of the successful construction of SNX31^m/m^ mice from DNA, mRNA, and protein levels.

**Figure 3:**
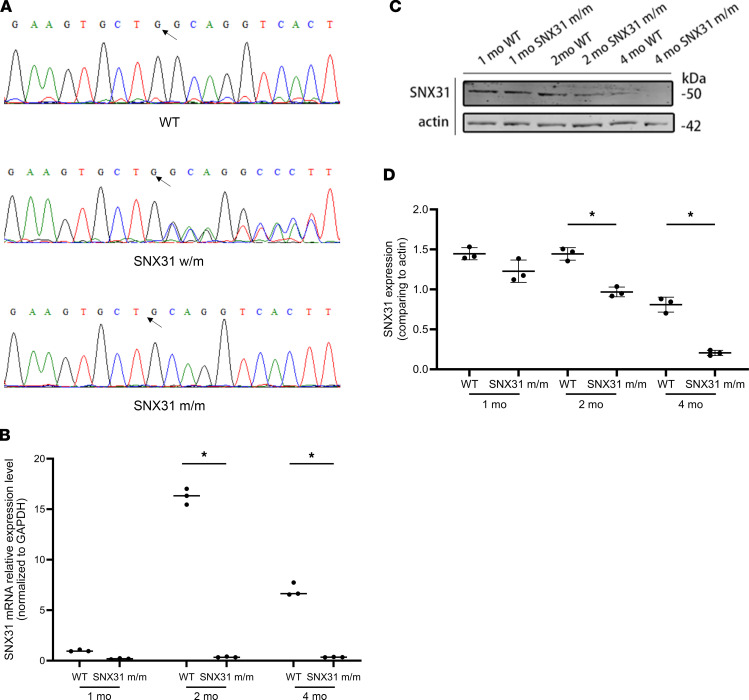
Validation of the construction of *SNX31^m/m^* mice from DNA, mRNA, and protein levels. (**A**) Genotype Sanger sequencing of WT, SNX31 heterozygous mutant mice (*SNX31^w/m^*), and *SNX31^m/m^* mice. WT: Sanger sequencing showed that the G base at position 963 of SNX31 was not deleted. SNX31w/m showed overlapping peaks, indicating a heterozygous deletion of G. SNX31m/m G base was successfully deleted and presented as a single peak, proving that the genotype was homozygous. (**B**) The relative expression of SNX31 mRNA in WT and *SNX31^m/m^* mice at different ages. The mRNA expression of SNX31 in *SNX31^m/m^* mice was significantly decreased. (**C** and **D**) The protein expression of SNX31 in WT and *SNX31^m/m^* mice at different ages. The expression of SNX31 in 2-month-old and 4-month-old *SNX31^m/m^* mice was significantly lower than that in the WT group. Statistical analysis was performed via unpaired parametric *t* test. Data are shown as mean ± SEM. m, mutant; mo, age in months; w, wild. *P < 0.05. See complete unedited blots in the supplemental material.

To construct the *SNX31* c.963delG mouse model, we used CRISPR/Cas9 technology to cut exon 10 of *SNX31* from zygotes of C57 strain mice. The homologous template with *SNX31* c.963delG point mutation was then repaired by DNA homologous recombination to achieve template base replacement at exon 10. Homozygous *SNX31* c.963delG mice were subsequently obtained by mating. The mutation was identified from DNA, mRNA, and protein levels, respectively. According to the sequencing results (Figure 3A), it can be seen that guanine was successfully knocked out at the 963 residue of SNX31.

To verify the knockin efficiency at the mRNA level, we extracted mRNA from retinal tissues of *SNX31^m/m^* mice and performed real-time quantitative PCR analysis (Figure 3B). Compared with that in WT mice of the same age, the expression of *SNX31* in mutant mice was significantly decreased, and this downward trend was particularly evident by the beginning of 2 months of age.

After extracting retinas from *SNX31^m/m^* mice, we performed Western blotting (Figure 3, C and D). The expression of SNX31 in 2-month-old and 4-month-old mice was significantly lower than that in the WT group, which indicated that the *SNX31* c.963delG homozygous mouse (*SNX31^m/m^*) was successfully modeled.

### Vascular leakage and retinal atrophy were observed in the fundus of SNX31^m/m^ mice.

**Figure 4:**
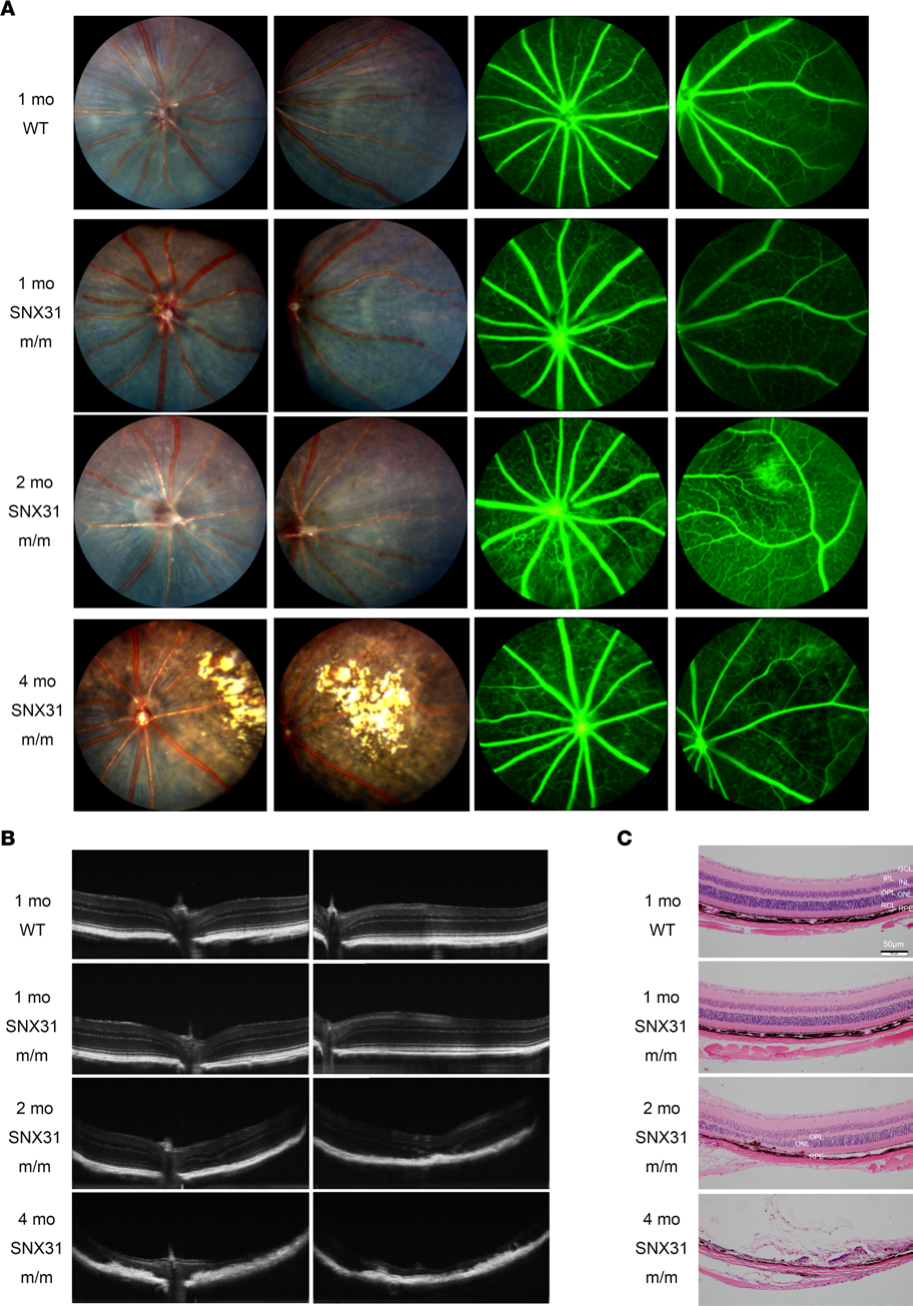
Vascular leakage and retinal atrophy are observed in the fundus of *SNX31^m/m^* mice. (**A**) Fundus photography and angiography of WT and 1-, 2-, and 4-month-old *SNX31^m/m^* mice. (**B**) OCT results of the optic disc and paraoptic retinas of WT and 1-, 2-, and 4-month-old *SNX31^m/m^* mice. (**C**) H&E staining of WT and 1-, 2-, and 4-month-old *SNX31^m/m^* mice. GCL, ganglion cell layer; INL, inner nuclear layer; IPL, inner plexiform layer; ONL, outer nuclear layer; OPL, outer plexiform layer; RCL, rod and cone layer. Scale bars: 50 μm.

The fundus of C57BL/6J WT and *SNX31^m/m^* mice was normal at 1 month of age (Figure 4A), as the FFA showed uniform distribution of retinal blood vessels and no abnormal blood vessel morphology.

However, at the age of 2 months, *SNX31^m/m^* mice began to have aberrant retinal blood vessels, manifested as delayed perfusion of retinal blood vessels or microvascular leakage, and obvious hypoperfusion occurred. As the disease continued to progress, *SNX31^m/m^* mice began to show focal or diffuse atrophy of retinal pigment epithelium (RPE) at about 4 months of age, corresponding to patchy penetrating hyperfluorescence foci on fundus fluoroscopy; at the same time, because of the absence of microvessels and large areas of nonperfusion, anastomosis of the great retinal vessels and pale atrophy of the optic nerve occurred.

We also performed optical coherence tomography (OCT). The structures of the inner and outer retinal layers of 1-month-old WT and *SNX31^m/m^* mice were normal, without abnormally high or low reflectance signals or structural disturbances (Figure 4B). As the *SNX31^m/m^* mice aged, different degrees of retinal structural disorder appeared. At 2 months of age, the retinal ellipsoid band was discontinuous, the outer nuclear layer showed focal thinning, focal thickening and mass-like hyperplasia were seen in RPE, the retinal outer nuclear layer was thinning or even disappeared in some parts, and the number of retinal layers was unclear. At 4 months of age, only the remaining retinal tissue near the optic disc was visible, in a state of severe retinal atrophy. It was accompanied by hyperreflective signals of large blood vessels, near disappearance of the inner and outer layers of the retina, RPE hyperplasia, and extensive scarring. The results of OCT were consistent with those of FFA: mottled, hyperintense focal hyperplasia of RPE, corresponding to extensive pigmentation on retinal color fundus photographs and foci of fluorescent shadows on angiography. The OCT results also showed that, in addition to the leakage of retinal vascular fluorescein, retinal thinning was one reason for the uneven background fluorescence of the retina on FFA examination.

To further observe the changes in retinal tissue in each layer of *SNX31^m/m^* mice, we embedded the eyeballs of WT and *SNX31^m/m^* mice in paraffin and stained them with H&E. Consistent with OCT, there were no abnormalities in the inner and outer retinal layers in WT and *SNX31^m/m^* mice at 1 month of age (Figure 4C). At 2 months of age, *SNX31^m/m^* mice had different degrees of retinal atrophy with a focal distribution, more pronounced near the optic disc. H&E staining showed that the outer plexiform layer and outer nuclear layer were obviously thinned, and the structure of the cone-rod layer was disorganized. The RPE was disrupted, proliferated, and migrated into the inner retinal layers. At the age of 4 months, the H&E staining of most mutant mice showed that RPE proliferated and migrated into adjacent retinal structures. The boundaries of the retinal layers were unclear, and the inner and outer nuclear layers almost disappeared, filled with a large number of abnormal cells and debris. The results of H&E and OCT correlated, and the retinal structure of *SNX31^m/m^* mice gradually became thinner and displayed atrophy. An early manifestation was focal outer neuroretinal thinning, mainly the outer nuclear layer and outer plexiform layer. With the destruction of the cone and rod layer, it gradually expanded to the inner nuclear layer and nerve fiber layer, until the formation of a large area of retinal structural disorder involving RPE and full-thickness retinal atrophy occurred.

### Blood vessels of SNX31^m/m^ mice are defective.

**Figure 5:**
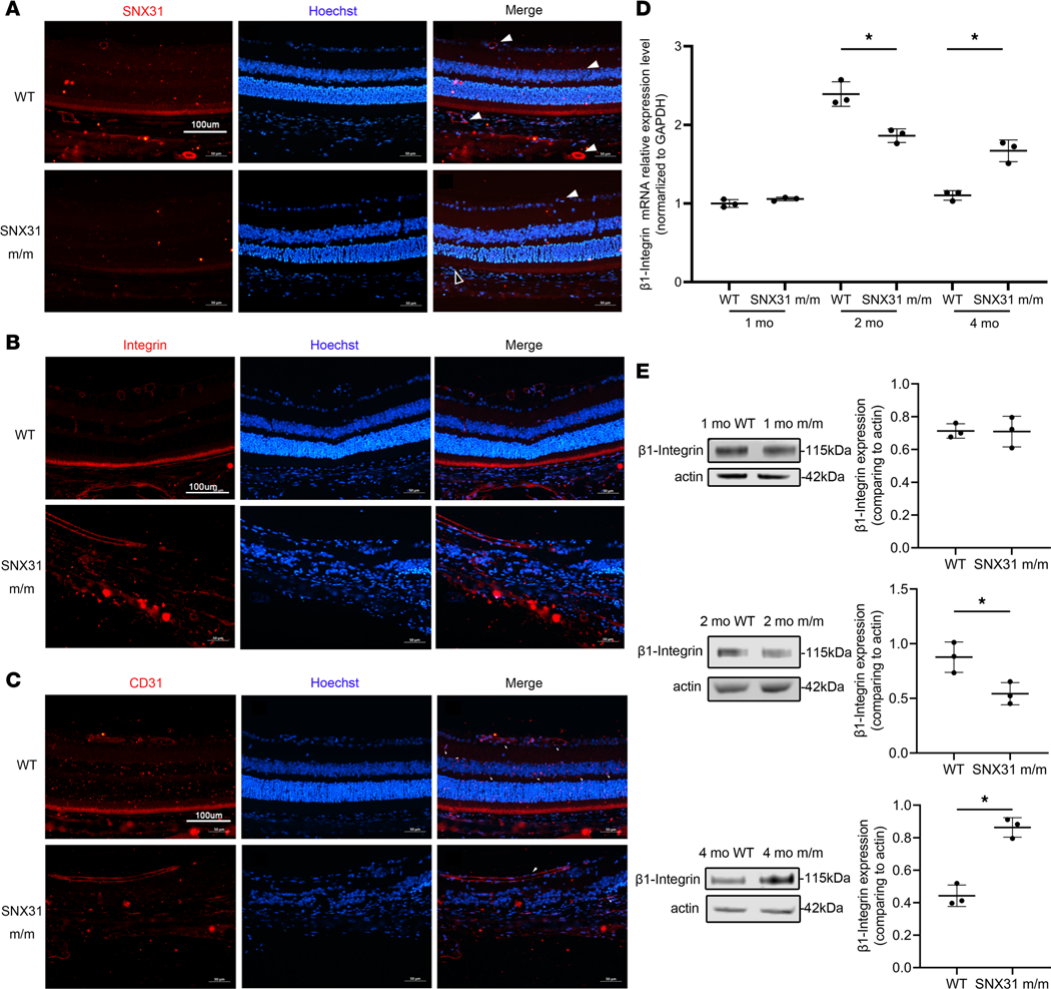
Immunofluorescence staining of SNX31, β1-integrin, and CD31 in retinas of WT and *SNX31^m/m^* mice. (**A**) SNX31 expression in retinal sections of 2-month-old WT and *SNX31^m/m^* mice. SNX31 fluorescence signal localized around blood vessels. White arrows show the fluorescent signal of SNX31; black arrows indicate RPE disruption and thinning of the outer nuclear layer above. Red, SNX31; blue, Hoechst-stained nuclei. (**B**) β1-integrin expression in retinal sections of 2-month-old WT and *SNX31^m/m^* mice. Red, β1-integrin; blue, Hoechst-stained nuclei. (**C**) CD31 expression in retinal sections of 2-month-old WT and *SNX31^m/m^* mice. Red, CD31; blue, Hoechst-stained nuclei. (**D** and **E**) Comparison of retinal β1-integrin mRNA and protein expression in WT and *SNX31^m/m^* mice at 1, 2, and 4 months of age. Statistical analysis was performed via unpaired parametric *t* test. Data are shown as mean ± SEM. *P < 0.05. See complete unedited blots in the supplemental material.

Immunofluorescence staining was used to explore the localization and expression of SNX31 in the retina (Figure 5A). We can see that the signal of SNX31 is mainly around blood vessels. At the same time, SNX31 was significantly less abundant in *SNX31^m/m^* mice compared with age-matched 2-month-old WT mice. Because we found that the mutant SNX31 protein cannot interact with β1-integrin in cellular experiments (Figure 2B), we also performed immunofluorescence staining for β1-integrin (Figure 5B). The β1-integrin fluorescence signal of WT mouse retina was distributed mainly around retinal blood vessels and the retinal cone-rod cell layer and RPE layer. In the retina of *SNX31^m/m^* mice, however, the distribution of β1-integrin signal near retinal blood vessels showed a downward trend, and the residual signal was concentrated mostly in the superficial dilated blood vessels. The β1-integrin located in the RPE and cone-rod cell layer also weakened and interrupted the fluorescence signal through RPE damage. Given the vascular damage seen in the previous experiments, we performed immunostaining by using the well-known vascular marker CD31 to analyze changes in vascularity in the mouse retina (Figure 5C). We can see that in the retinal structure of *SNX31^m/m^* mice, the signals of the middle and deep retinal capillaries that should be present are not clear. Only the retinal superficial large vessels were seen dilated.

Subsequently, we explored the mRNA and protein expression changes of β1-integrin in retinas of WT and *SNX31^m/m^* mice at different ages (Figure 5, D and E). At 1 month of age, the mRNA and protein expression levels of β1-integrin were not statistically different between *SNX31^m/m^* mice and WT mice. However, there was a significant difference in β1-integrin at 2 and 4 months of age, and it showed a trend of first decreasing and then increasing.

The expression of β1-integrin decreased, perhaps because of the decrease in SNX31, but the later increase of expression puzzled us. Therefore, we investigated the expression and distribution of SNX17, the same subfamily molecule with the highest homology to SNX31. It can be seen that the expression of SNX17 in the retinas of *SNX31^m/m^* mice aged 1, 2, and 4 months was higher than that of WT mice of the same age (Supplemental Figure 1; supplemental material is available online at https://doi.org/10.1172/jci.insight.167032DS1). Subsequently, we found that SNX17 was located mainly in the nerve fiber layer of WT adult mice on immunofluorescence staining (Supplemental Figure 1). However, similar to the Western blotting results, *SNX31^m/m^* mice showed significantly higher SNX17 expression, covering almost the entire retina. Thus, our results indicate that the deep retinal capillary network is impaired in *SNX31^m/m^* mice, which also confirms the nonperfused zone of FFA. The occurrence of these phenotypes may be related to the decrease of β1-integrin. On the other hand, the late increase may be related to the compensatory increase in SNX17 expression.

### VEGF pathway–related molecules may be involved in retinal vascular defects in SNX31^m/m^ mice.

**Figure 6:**
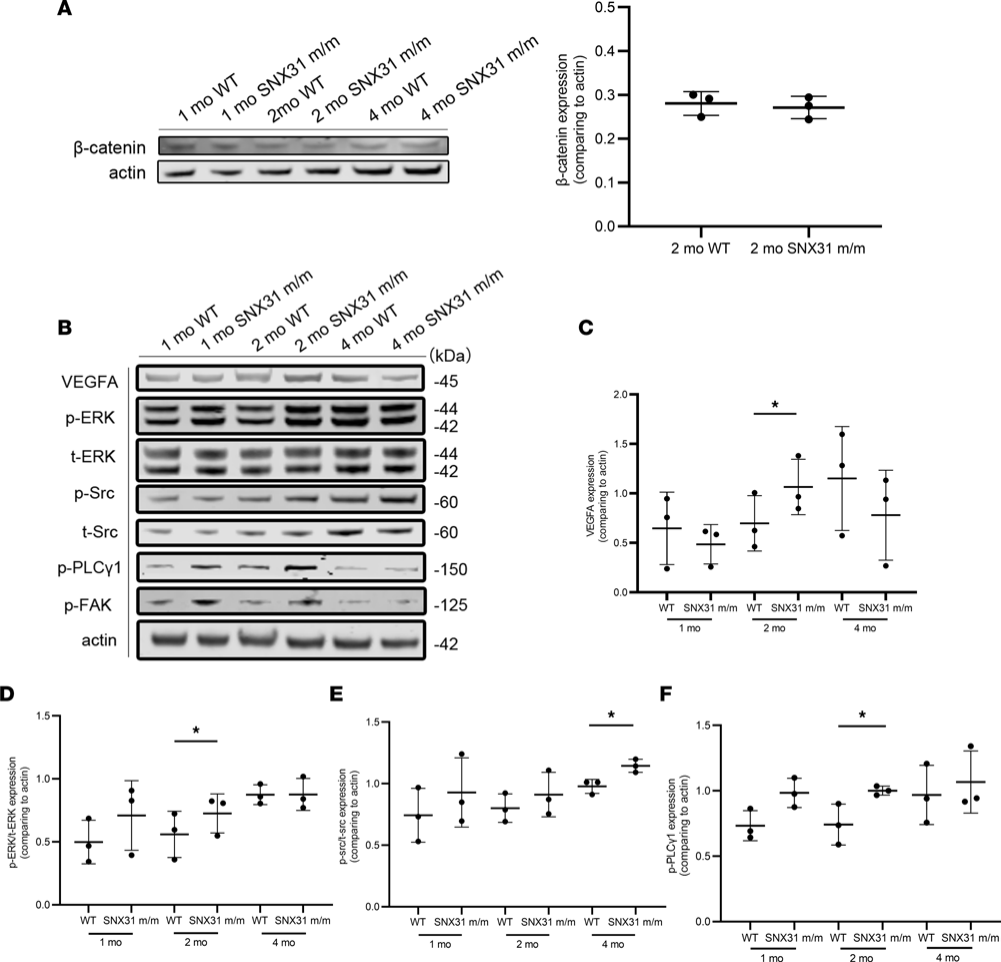
VEGF pathway–related molecules may be related to retinal vascular defects in *SNX31^m/m^* mice. (**A**) The expression of β-catenin in retinal tissues of mice of different ages. No obvious activation or inhibition trend was found for β-catenin. (**B**) VEGFA, p-ERK, t-ERK, p-Src, t-Src, p-PLCγ1, p-FAK, actin in retinas of 1-, 2-, and 4-month-old WT and *SNX31^m/m^* mice. The blots are not from the same lane, but the rest of the conditions are the same. (**C**) VEGFA increased at 2 months and decreased at 4 months. (**D**) p-ERK/t-ERK increased at 2 months. (**E**) p-Src and t-Src increased at 2 months, and the increase was more obvious at the age of 4 months. (**F**) p-PLCγ1 increased at the age of 2 months. P-ERK, phosphorylated ERK; p-FAK, phosphorylated FAK; p-PLCγ1, phosphorylated PLCγ1; p-Src, phosphorylated Src; t-ERK, total ERK; t-Src, total Src. Statistical analysis was performed via unpaired parametric *t* test. Data are shown as mean ± SEM. *P < 0.05. See complete unedited blots in the supplemental material.

To explore whether the known FEVR pathogenic pathway Wnt/β-catenin or the Norrin/β-catenin pathway is involved in the induction of fundus phenotypes in *SNX31^m/m^* mice, we detected the expression of β-catenin, the core protein of these two pathways, in retinal tissues of mice at different ages (Figure 6A). No obvious activation or inhibition trend was found for β-catenin, suggesting that the β-catenin pathway may not play a central role in retinopathy in *SNX31^m/m^* mice.

There is a lot of overlap between the downstream pathway of VEGF and the integrin pathway, and some integrins can directly bind to VEGF (20). We explored the VEGF-related pathways in *SNX31^m/m^* mice, considering retinal leakage and ischemic status (Figure 6B). The results showed that, in the retinas of *SNX31^m/m^* mice, VEGFA was significantly upregulated when vascular leakage began to appear at 2 months of age but decreased significantly at 4 months of age during the retinal atrophy period (Figure 6C). The upregulation trend of p-ERK was statistically significant only in 2-month-old mice (Figure 6D). p-Src showed a trend of upregulation in 2-month-old mouse retinas and was more pronounced in 4-month-old mice, with full-thickness atrophy involving RPE in the retina (Figure 6E). Likewise, p-PLCγ1 was upregulated statistically significantly only in the retina of 2-month-old mice (Figure 6F).

These results indicated that VEGF pathway–related molecules tended to be significantly upregulated in *SNX31^m/m^* mice in the period of vascular leakage at 2 months of age. However, in the period of retinal atrophy at the age of 4 months, the trend of VEGF pathway–related molecules was different.

### Retinal atrophy in SNX31^m/m^ mice is associated with activation of apoptosis-related pathways.

**Figure 7:**
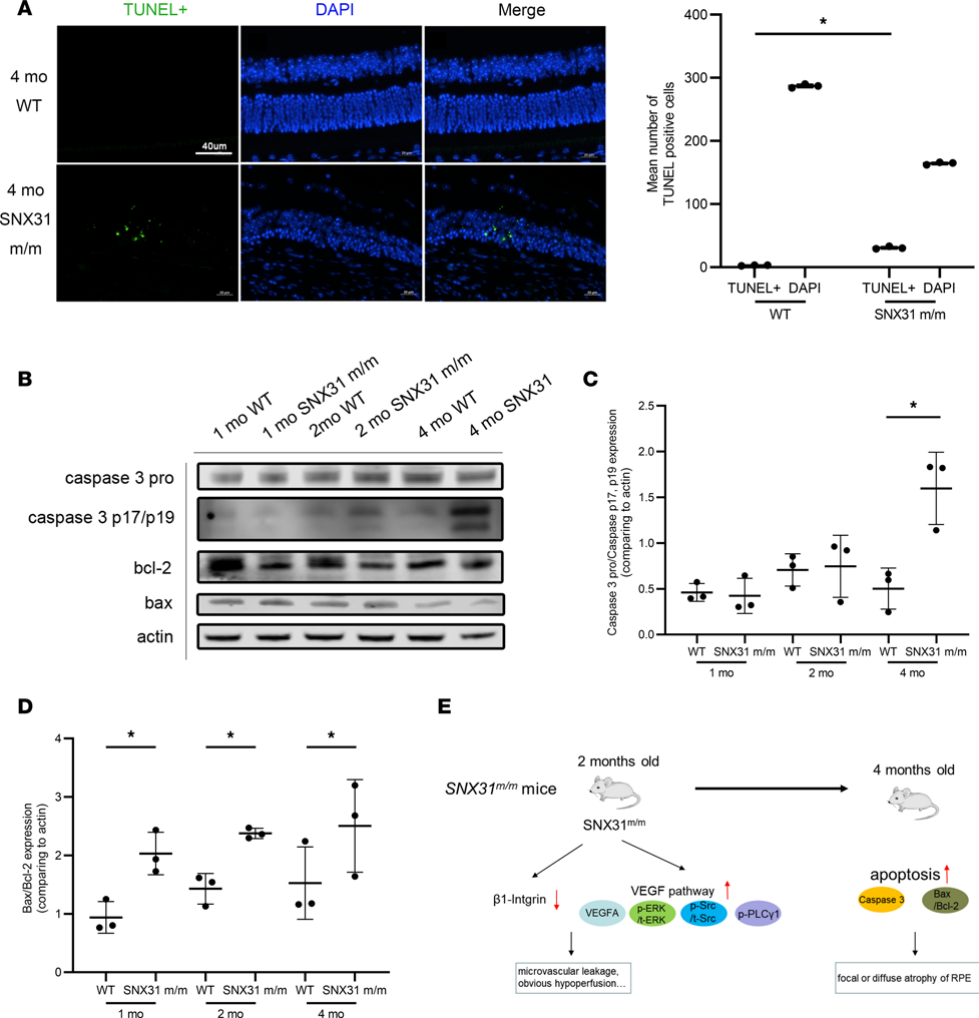
Retinal atrophy in *SNX31^m/m^* mice is associated with activation of apoptosis-related pathways. (**A**) Retinal TUNEL staining and statistical analysis results of 4-month-old WT and *SNX31^m/m^* mice. Green fluorescence, TUNEL-positive cells; blue fluorescence, DAPI. Original magnification, ×40. (**B**–**D**) Western blotting and statistical analysis results of retinal apoptosis-related proteins in WT and *SNX31^m/m^* mice aged 1, 2, and 4 months. Both caspase-3 and Bax/Bcl-2 were upregulated in 4-month-old *SNX31^m/m^* mice. The blots are not from the same lane, but the rest of the conditions are the same. (**E**) A brief mechanism diagram of the fundus phenotype of *SNX31^m/m^* mice. Statistical analysis was performed via unpaired parametric *t* test. Data are shown as mean ± SEM. *P < 0.05. See complete unedited blots in the supplemental material.

In addition to abnormal retinal blood vessels, *SNX31^m/m^* mice also developed retinal tissue atrophy lesions at 4 months of age. We therefore performed TUNEL immunofluorescence staining of retinas of 4-month-old WT and *SNX31^m/m^* mice (Figure 7A). As seen in TUNEL staining of *SNX31^m/m^* mice, TUNEL stained a larger number of cells than in WT mice. These cells are distributed mainly in the outer nuclear layer of the retina, and a small number of cells and cell fragments are distributed in the inner nuclear layer. The results of TUNEL immunofluorescence indicated that apoptosis-related pathways were an important mechanism of retinal atrophy

Western blotting results of apoptosis-related molecules were also consistent with TUNEL immunofluorescence staining (Figure 7, B–D). Caspase-3 showed an obvious activation trend in the retinas of 4-month-old *SNX31^m/m^* mice; at the same time, Bax/Bcl-2 also showed an obvious upward trend in the 4-month-old group. These results demonstrate that apoptosis-related molecules play an important role in retinal atrophy in mice.

## Discussion

In this report, we identified a possibly new *SNX31* variant that contributes to FEVR pathogenesis in a Chinese pedigree. Biochemically, we demonstrated that this variant fails to bind integrin and leads to integrin instability. Through cellular assays, we showed that the variant is defective in HRMEC tube formation, cell migration rates, and cell proliferation. Our work thus expands the mutation spectrum of FEVR to include *SNX31* and also suggests that integrin might be an overlooked factor of FEVR.

The clinical manifestations of each member of the family with FEVR were different. III:1 showed fluorescence leakage of retinal blood vessels during retinography, IV:1 showed mainly retinal avascular areas, and IV:2 showed mainly retinal folds. These clinical manifestations have obvious heterogeneity, which is consistent with the disease characteristics of FEVR. At present, the recognized pathogenic genes of FEVR include *FZD4* (21), *LRP5* (10), *NDP* (13), *TSPAN12* (22), *KIF11* (23), and *ZNF408* (24). The clinical manifestations are mainly retinal avascular areas, retinal vascular fluorescence leakage, and retinal detachment. At the same time, according to reports, the same gene mutation has different clinical manifestations, and the same clinical phenotype can also be derived from different gene mutations, with obvious genetic heterogeneity and phenotypic heterogeneity. These are similar to those of the SNX31 family we found.

As has been well documented, integrins are involved in a wide range of biological processes, including cell migration, adhesion, basement membrane formation, and control of the cell cycle (25, 26). It transmits intracellular information, supports vascular cells to establish new blood vessels that are suitable for the environment, mediates the interaction between cells and the extracellular matrix, and plays a key role in the development and distribution of blood vessels (27).

SNX31 belongs to the SNX protein family, and these proteins are involved in the transport of many proteins in the endosome system, such as P-selectin (28, 29), amyloid precursor protein (30, 31), integrins (32, 33), and members of the LDL receptor family (34, 35). After these proteins are endocytosed, the fate of receptors and transmembrane proteins is determined by specific endosomal sorting pathways, including cycling to the cell surface to remain active. It has been suggested that SNX31 can stabilize multivesicular body–associated plaques of urothelial umbrella cells (36). In 2014, studies found that mutations in the SNX31 gene can lead to schizophrenia (37). Moreover, the role of other family members of SNXs in the retina has been found in some studies. For example, zebrafish expresses and regulates SNX5 in the retina during early development to fine-tune Notch signaling to influence retinal development (38). SNX9-driven actin is reduced in human disease-associated oculocerebrorenal syndrome of Lowe deficiencies (39). SNX11 participates in retinal vascular development through F2R-like trypsin receptor 1 (40).

A role of β1-integrin in eye development is not unexpected. A study found that β1-integrin is significantly expressed in the retinas of mice after birth, especially in newly formed vascular buds (19). β1-Integrin is not only necessary for the germination of deep retinal vascular endothelial cells but is also essential for the formation of stable and mature blood vessels. In addition, it can also inhibit the activity of neovascularization, such as proliferation and germination (41). The phenotype of β1-integrin–knockout mice has many similarities to the fundus phenotype of clinical patients with FEVR. Integrin subunit β 1 mice show damaged retinal blood vessel formation, increased local retinal blood vessel density, peripheral retina local hemorrhage, instability of central vascular plexus capillaries, and deep retinal vascular sprouting defects (19).

In terms of disease mouse models, we completed the construction of *SNX31* c.963delG mutant mice by CRISPR/Cas9 technology. The homology of this SNX31 gene in humans and mice is 80.81%. The functional and histological examinations of the fundus phenotype were carried out, and the changes of SNX31 in the retinas of *SNX31^m/m^* mice were explored, as well as the molecular changes in other pathways involved in pathogenesis (Figure 7E).

As the disease progressed, we found that the fundus phenotype of *SNX31^m/m^* mice was manifested mainly in two aspects. One is the decline of retinal vascular stability, manifested as the above-mentioned retinal vascular leakage, delayed fluorescein filling, and even the formation of regional filling defects. The other is the thinning and atrophy of the retina that gradually begins to appear in the late stages. This degenerative change in the retina is manifested as focal outer neural retinal thinning in the early stage, mainly in the outer nuclear layer and the outer plexiform layer. With the destruction of the rod and cone layers, it gradually expanded to the inner nuclear layer and the nerve fiber layer, until the formation of full-thickness retinal atrophy involving RPE. In the severe cases, severe pan-retinal ischemia, anastomosis of retinal vessels, and pallor and atrophy of the optic disc can occur.

Considering the abnormal changes in retinal blood vessels in *SNX31^m/m^* mice, we first explored changes in VEGF-related pathways. VEGF is of great significance to the processes of angiogenesis and angiogenesis, and VEGF knockout can produce embryonic lethal effects (42, 43). At the same time, there is a lot of overlap between the downstream pathway of VEGF and the integrin pathway, and some integrins can directly bind to VEGF (20). At 2 months of age, retinal vascular abnormalities and areas of nonperfusion gradually began to appear. Molecules of VEGF-related pathways also began to increase, which may be an adaptive pathway activation response to chronic hypoxia in the retina. For 4-month-old mice that have begun to develop full-thickness retinal atrophy involving RPE, VEGFA is downregulated, and it is speculated that there may be 2 reasons: a decrease in the primary cells that produce VEGFA (glial cells) (44) and vascular compensation that occurs to alleviate the hypoxic state.

In the retinas of *SNX31^m/m^* mice, apoptosis-related pathways were activated at the later stage of vascular abnormalities, and apoptotic cells were located mostly in the inner and outer nuclear layers. The balance between ischemia and hypoxia and pathway compensation in retinal tissues other than full-thickness atrophy foci will determine whether the fundus phenotype of *SNX31^m/m^* mice embarks on a path of revascularization, stabilization, or further aggravation.

Meanwhile, there are still some differences between *SNX31^m/m^* mice and patients with FEVR, with peripheral retinal nonperfusion and microvascular leakage around nonperfused areas as major fundus symptoms, and onset before retinal vascular development is complete. We initially looked at the phenotypes of heterozygous mutant mice before breeding homozygous mice, but there was great variation within the population. Some heterozygous mice developed severe lesions such as reticular detachment at a very young age, whereas some mice never showed any symptoms. In contrast, the phenotypes of homozygous mice were more consistent. Therefore, in the selection of mice, in order to ensure the unity of the research, avoid the influence of accidental factors or unknown compensation of mutant mice, and better study the molecular mechanism of the gene, we chose homozygous mice. The disease-causing mutations in these patients with FEVR are heterozygous, whereas the animal models are homozygous, and there is a difference after all. This heterozygous mutation is relatively stable in humans, but it varies in severity in mice. We are currently conducting further research to investigate whether this is due to the differences between species. We speculate that the reason for the difference in FEVR phenotypes between *SNX31^m/m^* mice and humans is related to the following points. (a) Mutations in the same gene in humans may produce different phenotypes (e.g., NDP mutations can be associated with human FEVR, Coats’s disease, retinopathy of prematurity, and other retinal vascular diseases) (45). (b) There are interspecies differences, and (c) there are different stages in mechanisms that may affect angiogenesis or maturation. In their observation of endothelial-specific *Gab1*-knockout mice, Shioyama et al. found that the mutant mice did not have obvious defects in retinal angiogenesis during the developmental stage, but the neovascularization and revascularization stages were significantly impaired in response to lower extremity ischemic stress (46). These findings suggest that the molecular mechanisms involved in postnatal revascularization, maintenance of vascular stability, and abnormal angiogenesis under stress conditions such as hypoxia may be different from angiogenesis during normal embryonic vascular development. Finally, (4) other molecules of the same subfamily may compensate. The highly homologous SNX17 and SNX31 have some overlap in the types of proteins that can be recovered, and there may be a mutual compensatory mechanism. However, it remains to be determined at which stage *SNX17* and *SNX31* play a major role in angiogenesis.

In conclusion, *SNX31^m/m^* mice develop retinal vascular abnormalities, nonperfused areas, structural changes in the retina under ischemia-hypoxia, an autosomal dominant inheritance pattern, asymmetry of both eyes, skipping of the disease course, and great individual differences. We believe that the abnormal changes in retinal blood vessels caused by this mutation are very similar to FEVR (47). At the same time, this animal model also found that the *SNX31* point mutation leads to the appearance of a retinal vascular disease phenotype, indicating that SNX31 may play an important role in the maintenance of retinal vascular stability. At present, the mouse disease model constructed based on the discovered FEVR pathogenic gene is similar to our fundus symptoms. The superficial retinal vasculature of Lrp5-deficient rats was sparse and disorganized, with extensive exudates and decreases in vascularized area, vessel length, and branch point density (48). Fz4^−/−^ mice (45), Lrp5^P84L^ mice (49), and Jag1 endothelial-specific knockout mice (50) have a defect in vascular development or integrity.

Given our cellular and biochemical efforts and the construction of genetically mutant animal models, it is conceivable that the mutant *SNX31* inhibits the normal development of blood vessels. The most likely reason is that the normal cellular sorting function is lost and the β1-integrin cannot be cycled to the cell surface, where it supports vascular cells to establish new blood vessels suitable for eye development. Hence, our investigation implicates *SNX31* and integrin in FEVR pathogenesis and suggests that this could be a new therapeutic or diagnostic route for FEVR. The potential of this variant in other vascular-related disease, either systemic or ocular, may exist. Our study may provide scientific evidence of a potential genetic marker for future genetic screenings.

## Methods

*Pedigree and clinical assessment*. The family tree consists of 19 members from Shandong Province, located in eastern China. For clinical evaluation, clinical history surveys and eye examinations were carried out on the family members, including split-lamp examination ophthalmoscopy, fundus photographs, and fundus fluorescence photographs.

*Library preparation and whole-exome sequencing*. Genomic DNA was captured using the SureSelect Human All Exon v5 kit (51 Mb; Agilent Technologies). The Illumina NextSeq500 platform with 110 bp paired end reads was used to read independently for each captured library. The average sequencing depth of each sample was 100×. We used Illumina Pipeline (version 1.3.4) to process the original image file by using default parameters. Raw data were generated after whole-exome sequencing was completed. Regrettably, II:3 and III:2 did not undergo exome sequencing for various reasons.

*Plasmids*. *SNX31* and β1-integrin tail plasmids were cloned by reverse transcription PCR with human blood total RNA. All cloned constructs were confirmed by DNA sequencing. To generate HA-, FLAG-, and GST-tagged proteins, cDNAs were cloned in frame into CMV-HA (Clontech), p3XFLAG-Myc-CMV-26 (Sigma-Aldrich), and pGEX4t-1 (GE Healthcare) vectors, respectively. *SNX31* c.963delG was generated with a site-directed mutagenesis kit (Agilent Technologies).

*Antibodies*. Rabbit monoclonal antibodies against Flag were purchased from Abcam (1:5,000; ab2493). Mouse monoclonal antibodies against GST were obtained from Sigma-Aldrich (1:1,000; Novagen, 71097-3). Rabbit monoclonal antibodies against SNX31 were purchased from Biorbyt (1:500; orb592129). Horseradish peroxidase–conjugated AffiniPure secondary antibodies were purchased from Jackson (711-035-152, 115-035-116, 115-035-174).

*Cell culture and transfection*. HEK-293T cell lines and HRMECs were obtained from Angio-Proteomie. HEK-293T cell lines were grown in DMEM supplemented with 10% FBS and 1% penicillin and streptomycin (Invitrogen). HRMECs were grown in endothelial cell medium (ScienCell) containing 10% FBS, 1% endothelial cell growth factor, and 1% penicillin and streptomycin. Cells were transfected with various DNA plasmids by using FuGENE transfection reagents (Roche Applied Science) according to the manufacturers’ protocols. Cells were harvested for analyses at 24–48 hours after transfection.

*GST pull-down assays*. GST-tagged β1-integrin tail was expressed in BL21-CodonPlus (DE3)-RIPL cells (Agilent Technologies) and lysed by sonication and lysozyme treatment in lysis buffers. At the same time, we transfected WT *SNX31* and mutant *SNX31* in HEK293T cells, extracted the lysate, and interacted with the purified protein GST-integrin-β1-tail or GST-vec extracted as described above.

*Tube formation assay*. Matrigel basement membrane matrix (BD Biosciences) was thawed at 4°C, pipetted into precooled 35 mm Petri dishes, and incubated at 37°C for 1 hour. After Matrigel polymeration, cells were suspended in DMEM medium and were seeded onto the Matrigel. The experiments were carried out on HRMECs, including 4 groups including no transfection plasmid (blank control group), transfection of Flag-vec, Flag-*SNX31*-WT, and Flag-*SNX31*-Mut plasmids. For tube formation analysis, images were taken with a Nikon Eclipse microscope and operated by Spectrum software v. 2.8 (BD Bioscience) after 72 hours with the 10× objective.

*Transwell assay*. The Boyden chamber assay (NeuroProbe) was used to evaluate the migration ability of HRMECs. The experiment was performed in 24-well plates with Transwell inserts equipped with 0.8 μm pores coated with Matrigel at a 1:8 dilution (Becton Dickinson Labware).

*CCK-8 assay*. A CCK8 assay (Solarbio) was performed to examine the proliferation of HRMECs after transfection with WT or mutant *SNX31* plasmids. About 1 × 10^4^ cells were seeded into each 96-well plate and cultured in a 37°C constant temperature cell incubator containing 5% CO_2_ for 12 hours. Then, CCK8 solution was added for 45 minutes to perform absorbance measurement at a wavelength of 450 nm with a microplate reader (Molecular Devices).

*Generation of SNX31^m/m^ mice*. *SNX31^m/m^* mice were generated via the CRISPR/Cas9 nickase technique. The genomic RNA sequence was AAGCAACTGAGAATTTCTTGG. The donor oligo with the sequence GACATTGCTTTCCAGATGAGCAGAGTGAAGTGCTGCAGGTCACTTTCCTTGTGAGTATCTGGCAC was coinjected into the C57BL/6J zygotes to introduce an *SNX31* point mutation into the mouse genome. The *SNX31*-targeting allele was screened with a sequencing PCR product. Then, we hybridized *SNX31^+/m^* heterozygous mice to obtain *SNX31^m/m^* homozygous mice.

*Optical coherence tomography*. The pupils of the mice were dilated with compound tropicamide eye drops 10 minutes before the functional examination. Mice were anesthetized by intraperitoneal injection of 1.25% avertin (2,2,2-tribromoethanol) 0.02 mL/g. The cornea was coated with carbomer ophthalmic gel, and then OCT scans were taken at different positions of the retina.

*Color fundus photography and fluoroscopy*. Mice were dilated and anesthetized as above. The corneas of both eyes were coated with carbomer ophthalmic gel, the microscope lens of the small animal fundus imaging system was gently contacted with the cornea, and the focus and contrast were manually adjusted to show a clear fundus phase. After the fundus photography was completed, the mice were intraperitoneally injected with 1.7 mL/kg of 2% fluorescein sodium injection, and the above photography was repeated.

*Extraction of retinal proteins*. The retinas were separated and homogenized for 5 minutes on ice at a concentration of approximately 50 mg/mL in lysis buffer (Ready Prep Sequential Extraction Kit, Reagent 3, Bio-Rad Laboratories, Inc.). The protein concentration of sample was measured on a Model 550 Microplate Reader with a RC DC Protein Assay Kit (Bio-Rad Laboratories) with BSA as standard.

*Reverse transcription quantitative PCR*. Total RNA from mouse retina samples was extracted using TRIzol reagents (Invitrogen; Thermo Fisher Scientific Inc.). We used 1 μg total RNA as a template for cDNA synthesis with a Reverse Transcription system kit (Invitrogen; Thermo Fisher Scientific Inc.). PCR amplifications for the quantification of *SNX31*, β1-integrin, and GAPDH was performed with a SYBR Premix Ex Taq II (Perfect Real-Time) kit (Takara Bio) and an ABI PRISM 7300 Sequence Detection system (Applied Biosystems). GAPDH was used to normalize the relative mRNA expression levels of *SNX31* or β*1-integrin*, and then the 2^–ΔΔCt^ method was used to compare the expression levels. The primers used for reverse transcription quantitative PCR were as follows: *SNX31* forward primer, 5′-TATCACCATCCAGAATGTCGAG-3′; *SNX31* reverse primer, 5′-TCAGCCGTATCTGATGTTAGAC-3′; β*1-integrin* forward primer, 5′-TACTCTGGAAAATTCTGCGAGT-3′; β*1-integrin* reverse primer, 5′-ATAGCATTCACAAACACGACAC-3′; *GAPDH* forward primer, 5′-CGACTTCAACAGCGACACTCAC-3′; *GAPDH* reverse primer, 5′-CCCTGTTGCTGTAGCCAAATTC-3′.

*H&E staining*. Paraffin sections of the mouse retina were dewaxed with xylene, stained with hematoxylin for 1 minute, placed in eosin and stained for 1 minute, dehydrated in ethanol, and finally sealed with neutral resin and covered with coverslips. After solidification, the samples were examined under the microscope.

*TUNEL staining*. TUNEL staining was performed with a commercial in situ cell death detection kit, according to the manufacturer’s manual (Roche Diagnostics). In brief, the paraffin sections from the mouse retinas in each group were incubated with TUNEL reaction mix for 2 hours at 37°C and then counterstained nuclei with DAPI.

### Statistics

All results represent average values from 3 independent experiments. All error estimates are given as the mean ± SEM. The statistics were carried out via 1-way ANOVA and been calibrated via Tukey’s post hoc test. Statistical differences were determined via 2-tailed Student’s *t* test. *P* values of less than 0.05 were considered significant.

### Study approval

The study was performed in accordance with the provisions of the Declaration of Helsinki and approved by the Peking University People’s Hospital Ethics Committee. The protocol and all relevant study forms for the trial were approved by the Peking University People’s Hospital institutional review board (approval no. 2020PHE004).

## Author contributions

LH, JL, MZ, and XS contributed to the conception of the study; NX, YC, JL, TT, and CL performed the experiment; YS and NX contributed significantly to analysis and manuscript preparation; NX performed the data analyses and wrote the manuscript; LH, JL, LZ, MZ, and XL helped perform the analysis with constructive discussions. All authors reviewed and approved the manuscript.

## Supplementary Material

Supplemental data

## Figures and Tables

**Table 1:**
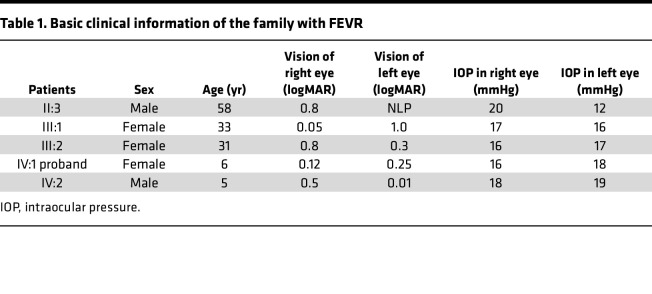
Basic clinical information of the family with FEVR

## Version 1 04/13/2023

In-Press Preview

## Version 2 05/22/2023

Electronic publication
