# Supplementary material for: An SNX31 variant underlies dominant familial exudative vitreoretinopathy-like pathogenesis
Source: JCI Insight. 2023 May 22;8(10):e167032. doi: 10.1172/jci.insight.167032 (PMC10322688; doi:10.1172/jci.insight.167032)

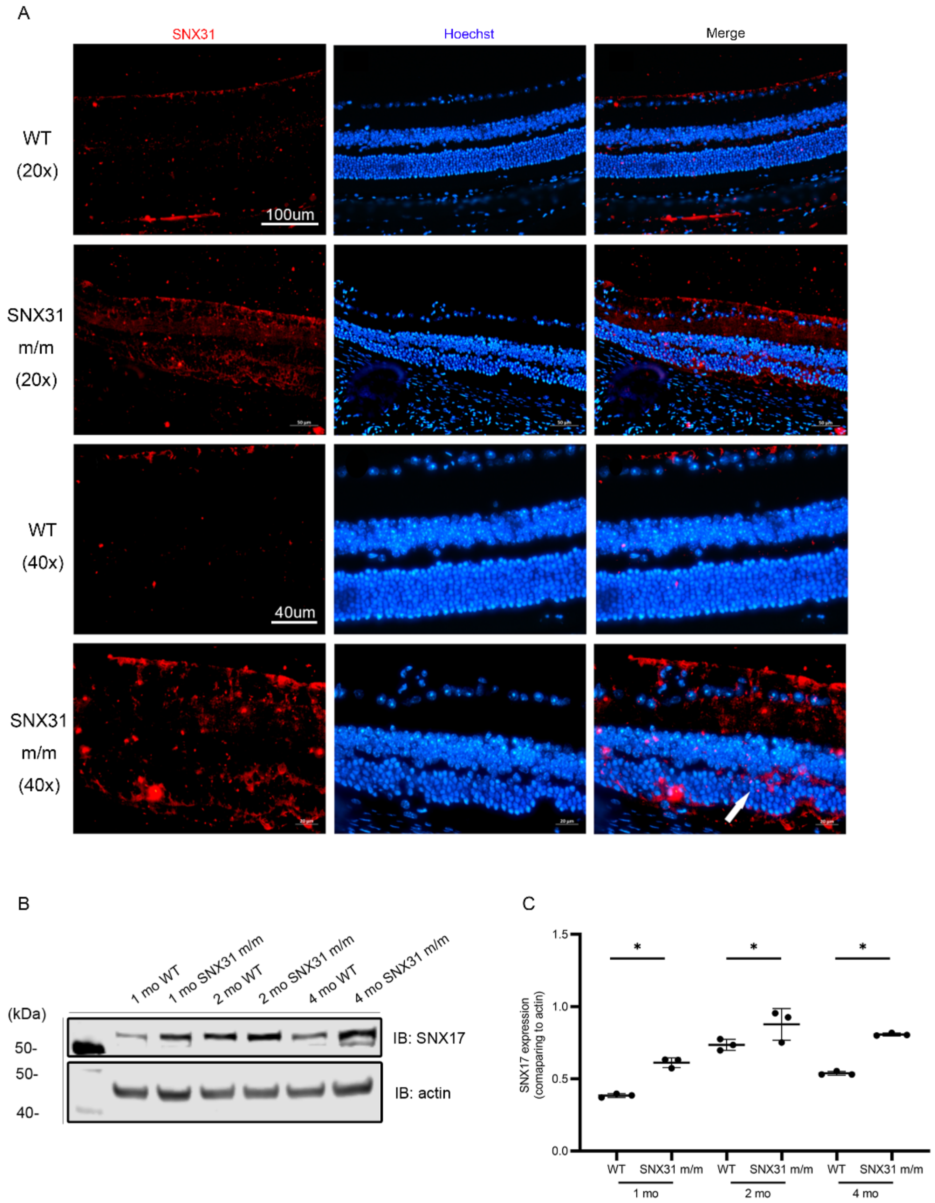

Figure S1. Immunofluorescence staining of SNX17 in retinas of WT and SNX31m/m mice. (A) The immunofluorescence results of SNX17 in WT and SNX31m/m mice. Increased expression of SNX17 in the outer plexiform layer is seen at the white arrow. Red: SNX17; blue: Hoechst-stained nuclei. (B) The protein expression of SNX17 was higher in SNX31m/m mice at 1, 2 and 4 months of age than in wild-type mice. Statistical analysis was performed using the unpaired parametric t test. Data are shown as mean ± SEM. *P<0.05.

Full unedited gel for Figure 2B:

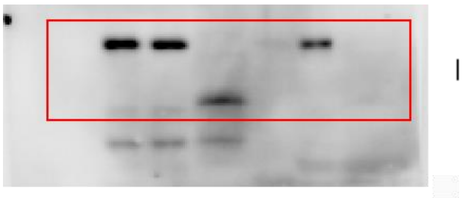

IB： HA

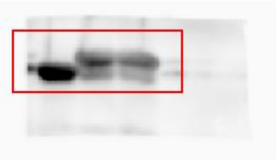

IB： GST

Full unedited gel for Figure 2C:

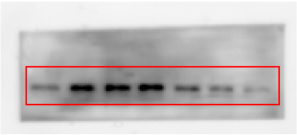

IB： β-integrin

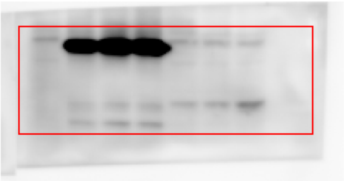

IB： Flag

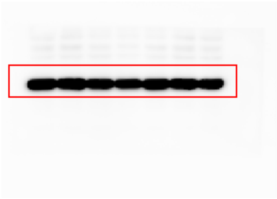

IB： actin

Full unedited gel for Figure 3C:

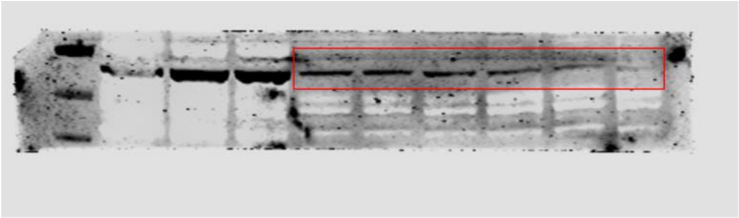

IB： SNX31

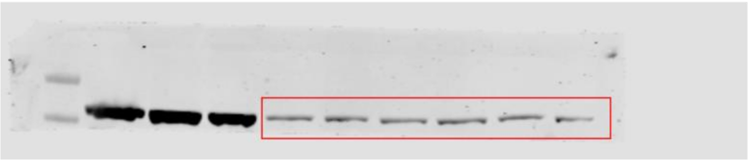

IB： actin

Full unedited gel for Figure 5E:

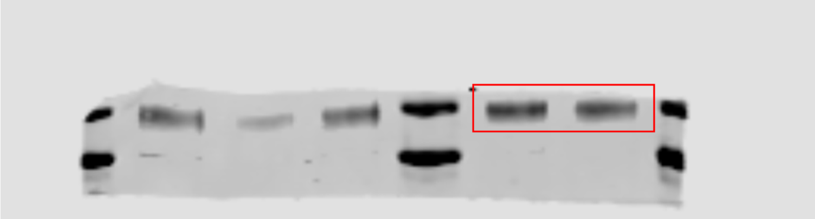

IB： integrin β1

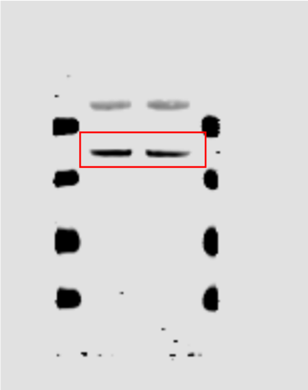

IB： actin

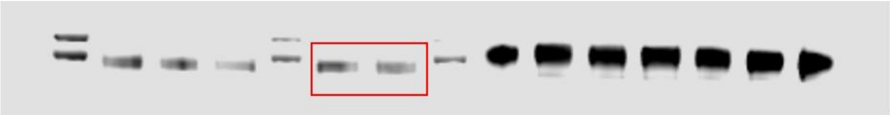

IB： integrin β1

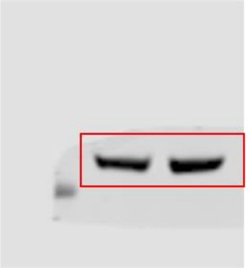

IB： actin

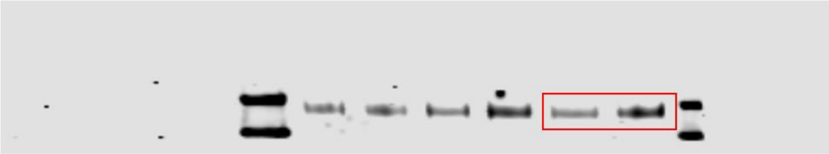

IB： integrin β1

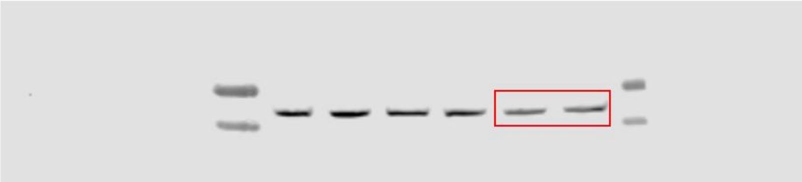

IB： actin

Full unedited gel for Figure 5F:

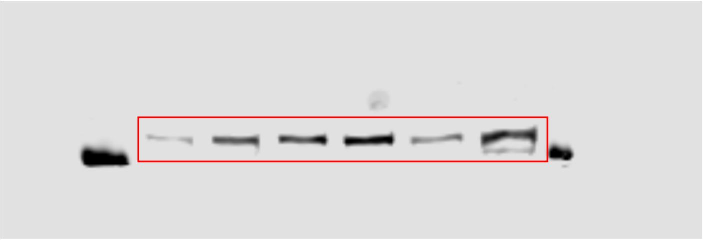

IB: SNX17

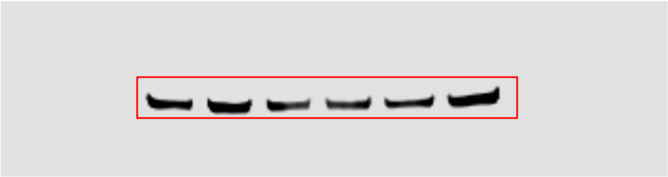

IB: actin

Full unedited gel for Figure 6A:

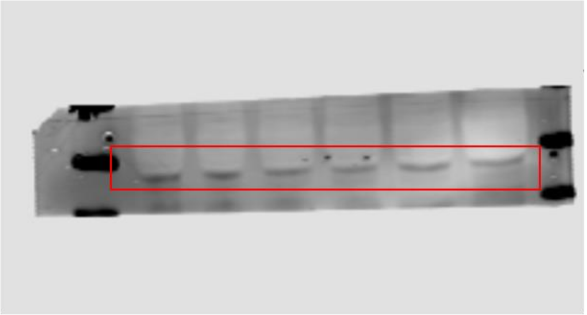

IB: β-catenin

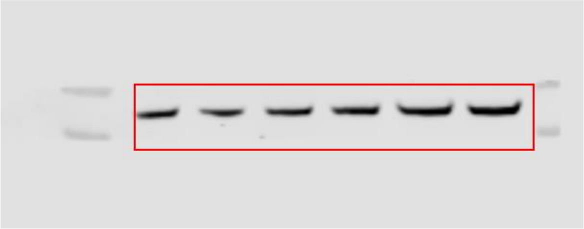

IB: actin

Full unedited gel for Figure 6B:

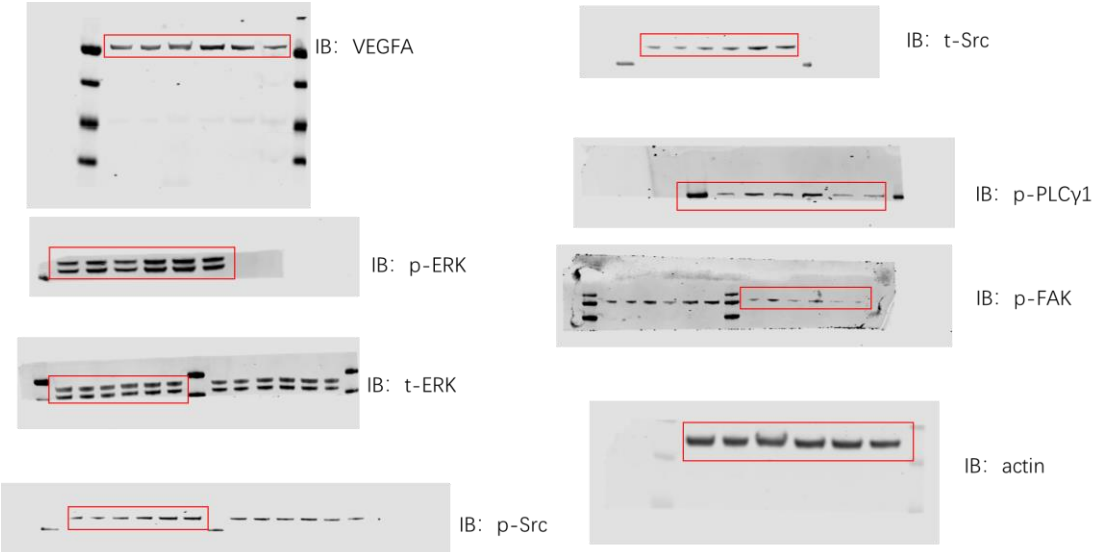

Full unedited gel for Figure 7B:

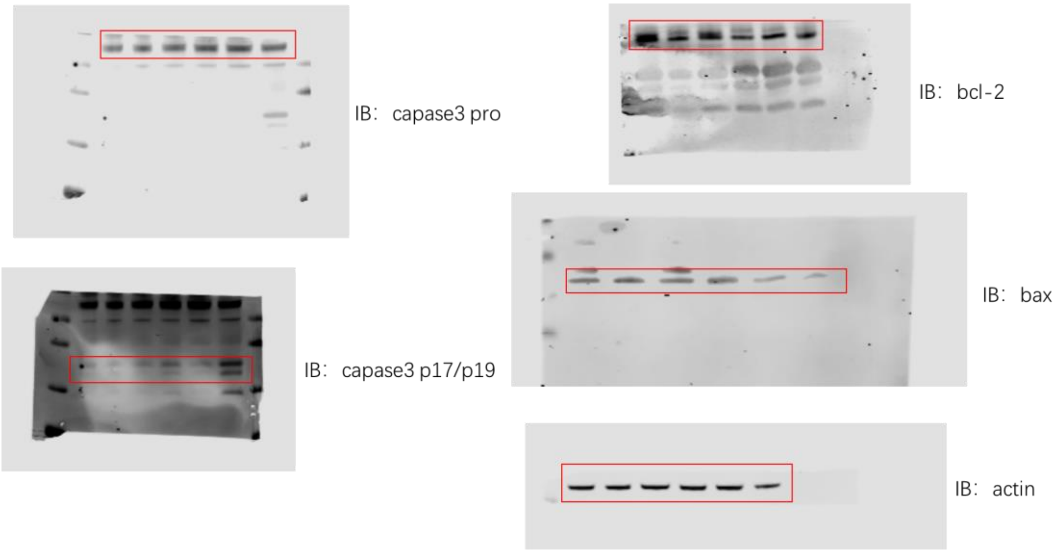

Supplement: Supplemental data [file jciinsight-8-167032-s160.pdf]
